# Editorial: Role of perceptual and motor representations in bilingual and second language processing

**DOI:** 10.3389/fnhum.2025.1555254

**Published:** 2025-01-24

**Authors:** Helen Zhao, Norbert Vanek, Jing Yang, Menghan Wang

**Affiliations:** ^1^School of Languages and Linguistics, The University of Melbourne, Parkville, VIC, Australia; ^2^School of Cultures, Languages and Linguistics, The University of Auckland, Auckland, New Zealand; ^3^School of International Studies, Zhejiang University, Hangzhou, Zhejiang, China

**Keywords:** mental imagery, perception, embodied cognition, motor action, bilingualism, second language processing, psycholinguistics, mental simulation

Much of language comprehension involves mentally simulating perceptions and actions we hear or read about. The articles in this Research Topic examine the extent to which bilinguals and second language (L2) learners mentally recreate experiences during L2 comprehension. Two common denominators characterize the studies. They strive not only to describe whether and how L2 learners engage embodied mental simulation when constructing meaning in their L2, but also to explore what it means to simulate native-like mental representations.

Wang and Zhao addressed the limitations of traditional sentence-picture verification tasks by employing image-schematic diagrams to investigate how bilingual speakers process spatial and abstract language in their L1 and L2. 41 L1 Mandarin speakers participated, with 21 completing tasks in Mandarin and 20 in translation-equivalent English, where they read sentences and judged the congruency of accompanying diagrams. The results showed robust mental simulation effects in L1 processing, unaffected by the type of concept, while L2 processing exhibited weaker effects, with interference only present for spatial concepts. These findings support the simulation-based L1 and L2 comprehension models, validate the sentence-diagram task paradigm, and stimulate further research on perceptual representations in bilingual language processing in areas beyond spatial features.

Vanek et al. examined how negation is processed in the L1 and L2 by analyzing eye movements on a blank screen during sentence comprehension. The innovative use of the blank screen paradigm enabled the researchers to capture different processing stages as they unfolded during language-modulated mental simulations. Thirty-two native Croatian speakers and 32 Croatian learners of English listened to sentences with various types of negation and affirmation in both languages. Eye movements were tracked to detect anticipatory looks toward expected visual information in the absence of actual images. The findings indicated that processing negation is more cognitively demanding than processing affirmation in both L1 and L2. Participants showed anticipatory eye movements in both languages, suggesting that listeners generate mental representations similarly across their two languages.

Wentura et al. investigated how proficient bilinguals process language through both sensory (modal) and abstract (amodal) representations, hypothesizing that L2 processing relies more heavily on abstract representations than L1 processing. The modality-switch task paradigm was employed, where 79 German-French bilingual participants evaluated noun-adjective pairs in both languages, judging whether the adjective appropriately described the noun. This method assessed the cognitive costs associated with switching between sensory modalities (e.g., from visual to olfactory attributes) versus maintaining the same modality. The results showed modality-switch effects (MSE) in L1 and L2 French, indicating that sensory-based processing extends to L2. However, a weak MSE in German L1 and no MSE in German L2 suggests that sensorimotor processes may not be obligatory in L2 processing.

Shiang et al. explored the role of motor embodiment during L2 sentence comprehension, focusing on how advanced Mandarin learners of L2 English mentally represent actions described in sentences. Eye-fixation data from both early and late eye-movement measures as well as reaction times were collected from 56 participants, who completed a sentence–picture verification task. Participants read action sentences in L2 and then viewed images depicting actions that either matched or mismatched the sentence content. Results showed longer response times and increased fixations on body effectors (e.g., hands) in mismatch conditions, particularly during later processing stages. These findings suggest that L2 learners engage in context-driven motor representations during sentence comprehension, supporting the embodied cognition framework in L2 processing.

Greenacre et al. examined how language structures influence magnitude comparison in Pitjantjatjara-English bilinguals. Pitjantjatjara, an Indigenous Australian language, lacks dedicated comparative constructions and has a limited set of lexicalized numerals, which was hypothesized to affect magnitude processing. Participants performed tasks comparing quantities of dots (numerosity) and lengths of lines (extent) in both languages on separate days, with accuracy and reaction times recorded. Findings showed no significant differences in numerosity comparisons between the languages. However, participants were less accurate in extent comparisons when using Pitjantjatjara, with accuracy decreasing as magnitudes increased and differences between items narrowed. This reveals that linguistic variation in expressing comparisons may influence the cognitive processing of specific magnitudes.

Chen et al. focused on how multilingual speakers mentally represent concepts in their first, second, and third (L3) languages. The researchers ran two experiments using a sentence-picture verification paradigm to assess perceptual representations in both working memory and long-term memory stages. Participants were presented with sentences in Cantonese (L1), Mandarin (L2), and English (L3), followed by images that either matched or mismatched the sentence content. Response accuracies and reaction times were analyzed. The findings indicated that perceptual representations were evident in L1 comprehension but were absent in L2 and L3, regardless of whether the task primarily engaged working memory or long-term memory. These findings suggest that perceptual grounding is more substantial in one's native language compared with later-learned languages, contributing to our understanding of embodied cognition in multilingual contexts.

Xue et al. took a developmental approach to embodied cognition and examined how sensorimotor experiences interact with the age of acquisition (AoA) of concepts in bilingualism, focusing on L1-Mandarin and L2-English bilinguals. Sensorimotor ratings were collected for 207 items, assessing the extent to which Mandarin-English bilinguals experienced these concepts through six perceptual senses and five action effectors. Data on AoA and word frequency were also gathered for both languages. The analysis revealed significant correlations between sensorimotor experiences and AoA in both languages. However, after controlling for word frequency, sensorimotor experiences explained additional variance in L1 AoA but not L2 AoA. This suggests that while sensorimotor grounding influences concept acquisition for both languages, its impact is more pronounced in L1.

Through seven complementary perspectives, this Research Topic highlights the need for nuanced experimental approaches and scope expansion to refine existing theories of embodied cognition across diverse bilingual and L2 contexts. Foregrounding the interdependencies of linguistic structures, mental models, cognitive demands, and developmental factors, these studies collectively demonstrate that bilingual language processing involves embodied and sensorimotor representations, with L1 often showing more perceptual grounding than later-acquired languages.

